# Buttermilk in Typhoid Fever

**Published:** 1910-07

**Authors:** 


### Buttermilk in Typhoid Fever.

Dr. Edward C. Register, of Charlotte, North Carolina, in a recent address on "Diet in Typhoid Fever," says: "We have no ideal food for the typhoid patients, neither have we any one food that will suit every case, or that is the best in every case; consequently, we have to select the food that seems to be most proper for the individual. Speaking generally, I am of the opinion that milk, in some of its forms, is the best diet. In fully 85 per cent of all the cases of typhoid fever that I have ever treated, milk seemed to be the best diet. For the last few years it has become a habit of mine in many cases to substitute sour milk or buttermilk for sweet milk. In the majority of cases I find that it is more palatable, and many of my typhoid patients prefer it. Buttermilk as an article of food in typhoid is not a new thing; its value has been known almost since the beginning of medical history, but it is only recently that we have fully learned to appreciate it as a food for fever patients. There is hardly any substance that has as great a tendency to check putrefaction as has sour milk, and at the same time is so easy to digest, and gives us a food of considerable nutritive value. I have observed evidences of bacterial putrefaction in patients to whom sweet milk was properly given, subside when the buttermilk diet was adopted."—*Exchange.*

